# Motor Coordination and Moderate-to-Vigorous Physical Activity in Emerging Adults: Mediating Effect of Physical Self-Concept

**DOI:** 10.3390/ijerph17113748

**Published:** 2020-05-26

**Authors:** Yao-Chuen Li, Daniele Chirico, Jeffrey D. Graham, Matthew Y. W. Kwan, John Cairney

**Affiliations:** 1 Department of Physical Therapy, China Medical University, Taichung 40402, Taiwan; yaochuenli@mail.cmu.edu.tw; 2 Department of Family Medicine, INfant and Child Health (INCH) Lab, McMaster University, Hamilton, ON L8S4L8, Canada; dchirico@totalcardiology.ca (D.C.); grahajd2@mcmaster.ca (J.D.G.); kwanmy@mcmaster.ca (M.Y.W.K.); 3 Faculty of Kinesiology & Physical Education, University of Toronto, Toronto, ON M5S2W6, Canada; 4 School of Human Movement and Nutrition Sciences, University of Queensland, Brisbane 4072, Australia

**Keywords:** adulthood, cross-sectional, mediation, motor behaviors

## Abstract

Little research has investigated the relationships among motor coordination, perceived physical self-concept (PSC), and physical activity during emerging adulthood. The purpose of this study was to investigate whether PSC mediates the relationship between motor coordination and moderate-to-vigorous physical activity (MVPA) in emerging adults. This was a cross-sectional study with 218 undergraduate students aged 17–23 years (167 females, 76.6%). Participants were asked to complete a questionnaire including self-reported measures of motor coordination, PSC and MVPA. The mediating effects of both overall and domain-specific PSC were tested on the relationship between motor coordination and MVPA. Motor coordination was found to have a significant indirect effect on MVPA through overall PSC. Exploratory analyses specifically showed a mediating effect of domain-specific PSC of activity on the relationships between motor coordination and MVPA during chores and leisure-time. Findings from the current study highlight the importance of PSC on the relationship between motor coordination and MVPA and showed that university students with poor motor coordination exhibit lower levels of PSC, specifically, the perception of activity. Future interventions targeting the enhancement of MVPA should focus on improvement in the self-perception of physical activity alongside motor skills training.

## 1. Introduction

There continues to be compelling evidence that physical activity is strongly and causally associated with a variety of physical and mental health benefits [1]. Risk estimates suggest that approximately 20%–30% of premature mortality could be prevented through regular engagement in physical activity [1]. Despite the many benefits, the majority of the North American population is not sufficiently active. In Canada, results from population-based studies suggest that only 15% of adults (individuals over the age of 18) meet the suggested 150 min of moderate-to-vigorous physical activity (MVPA) each week [2]. Thus, it is critical that we understand the underlying reasons for this, such as low physical activity engagement, which was found to be strongly associated with physical and mental health benefits [3]. 

Proficiency in motor skills was found to be an important correlate of physical activity for children and youth [4]. Studies consistently showed that school-aged children with motor impairments were less physically active compared to typically developing children, engaging in less free-play as well as organized activities [5]. Importantly, studies of children and youth across a broad range of motor skill abilities found positive associations between motor competence and physical activity engagement [4,6]. This could have significant implications developmentally, as motor competence, or lack thereof early in the lifespan, is hypothesized to persist into adulthood and lead to increased physical inactivity and low levels of fitness [7]. 

Psychological factors, such as enjoyment, self-efficacy, and motivation, are also well-documented correlates of physical activity across the early life course [8,9,10,11]. Among these, physical self-concept (PSC), which is considered as one of the dimensions of self-concept and specifically related to the perceptions of an individual’s physical abilities, gained research attention over recent years [12]. More importantly, when dividing PSC further into various subdomains, results from a recently published meta-analysis suggested that compared to composite or global measures of PSC, the subdomains of perceived competence, fitness, and appearance are more strongly correlated with physical activity behaviors during childhood and adolescence [13], highlighting the need to investigate not only the role of overall PSC, but also domain-specific PSC in physical activity research. 

It is also important to know that the evidence is robust with respect to the association between motor coordination and PSC. Lower motor coordination or poor motor skills were consistently found to be associated with more negative reporting of PSC [14,15]. Specifically, children or adolescents with motor difficulties consistently reported lower level of perceived athletic or sporting competence than typically developing children [16,17].

Together, these findings suggest a potential mediating pathway between motor coordination and physical activity through PSC [18]. However, this hypothesis has not been well examined, and there are still gaps in our knowledge. First, among the limited research examining motor coordination alongside psychological factors and physical activity, the population of interest has typically included children and adolescents [13,18,19]. Importantly, the transition out of high school and into emerging adulthood was identified to be a critical point when significant declines in leisure-time physical activity occur [20]. Few studies, however, have investigated the role of motor coordination in understanding physical activity behaviors during this emerging adulthood period; to date, no studies have attempted to understand the role that PSC may play in this relationship during this developmental period. In addition, previous research on emerging adulthood focused on overall patterns of physical activity [20] or specific forms of participation, such as sports [18,21]. While there are health benefits associated with even light forms of physical activity, the evidence for health benefits is stronger when the intensity is moderate to vigorous [3]. Therefore, in this paper, we not only focused on MVPA as the primary outcome, but also investigated MVPA across different domains of participation. 

Taken together, the primary purpose of this study was to examine the interrelationships among motor coordination, PSC, and physical activity during the emerging adulthood period. In general, it was hypothesized that lower levels of motor coordination would be associated with lower levels of PSC and physical activity, and that there would be a positive association between PSC and physical activity. Secondly, the mediating effect of PSC on the relationship between motor coordination and MVPA was examined. We hypothesized that, overall, PSC would mediate the effect of motor coordination on MVPA. 

Finally, when the relationships between physical activity subdomains and mental health were investigated in previous research, different physical activity subtypes showed various associations with psychological distress; for example, higher leisure-time physical activity was found to be associated with less psychological distress, whereas this negative association was not found for transport physical activity [22]. This may imply that the relationship between PSC and MVPA differs according to their domains. Therefore, in order to better understand these relationships in emerging adults with poor motor coordination, this study conducted an exploratory analysis by examining the potential parallel mediating effect of each domain-specific PSC simultaneously on the relationship between motor coordination and MVPA. 

## 2. Materials and Methods

### 2.1. Participants and Procedures

The study utilized a cross-sectional study design and enrolled undergraduate students aged 17 to 23 years (*n* = 218, mean age = 19.5 ± 1.0 years) from a mid-size university in the southern region of Ontario, Canada. All participants were recruited through study advertisements placed around campus and posted on social media. A screening email was first sent to students who showed interest in participating to confirm their eligibility by asking whether they had any neurological diseases or physical impairments which might account for poor or low scores on the motor coordination scale and negatively impact participation in physical activity. Eligible participants were then provided with a secure online link to access a survey which included the consent form, demographic and anthropometric information, and the questionnaires regarding their motor coordination, PSC, and MVPA. Ethical approval was obtained from a local Institutional Research Ethics Board, and all participants provided informed consent.

### 2.2. Measures 

Physical activity. Physical activity was measured using the International Physical Activity Questionnaire-Long Form (IPAQ-LF), which is a 7-day recall self-reported questionnaire [23]. Participants were asked to report their frequency and time spent in different activities (i.e., job-related, transportation, chores, and leisure-time physical activities) at a variety of intensities (e.g., walking, moderate, or vigorous) in the past seven days. According to the scoring protocol, each type of activity corresponded to a metabolic equivalent (MET), for example, moderate job-related physical activity was 4.0 METs, whereas vigorous job-related physical activity was 8.0 METs. Then, the MET minutes per week (MET-min/week) from each activity was calculated using the formula MET * average minutes per day * days a week. Consistent with physical activity guidelines, we included total MVPA as the primary outcome of interest. Further, we calculated the MET-min/week for each MVPA subdomain (i.e., job-related, transportation, chores, and leisure-time). The IPAQ-LF showed good test-retest reliability and concurrent validity when compared against accelerometry [24]. It was also found to exhibit good internal consistency in an Italian sample (α’s = 0.73) [25].

Motor coordination. The Adult Developmental Coordination Disorder/Dyspraxia Checklist (ADC) was used to assess motor coordination [26]. The 40-item ADC consists of two subscales that ask adolescents or adults to report the frequency (i.e., never, sometimes, frequently, and always) of childhood motor difficulties (Section 1), and current difficulties with motor tasks or activities of daily living (e.g., writing neatly, bumping into things, or avoiding team sports) (Section 2). The total score is the sum of the two subscale scores, with higher scores indicating poorer motor performance. Since the present study was interested in current motor functioning, only the subscale score of Section 2 was used as an indicator of participants’ motor coordination. The ADC was previously validated and demonstrated excellent internal consistency for the subscales (α’s = 0.87–0.91) [26]. 

Physical self-concept. The short form of the Physical Self-Description Questionnaire (PSDQ-S) was used to measure PSC [12]. The 40-item PSDQ-S measures eleven domains of PSC, including health, coordination, activity, body fat, sport, global physical, appearance, strength, flexibility, endurance, and global esteem [12,27]. Participants were asked to read each statement and choose the answer that best reflected their situation. The items were scored on a six-point Likert scale ranging from 1 (false) to 6 (true). The scores of each item were then added together to create the subscale; total scores with higher scores indicated higher PSC. The PSDQ-S previously demonstrated good internal consistency (α’s = 0.81–0.91) [12].

### 2.3. Data Preparation and Analysis

All statistical analyses were conducted using SPSS 22.0 for Windows (IBM Corp, Armonk, NY, USA). The sample size calculation for the proposed mediating effect of PSC was based on Kline (2015), suggesting that at least 5 to 10 participants were needed for each parameter [28]. Accordingly, an original sample size of *n* = 225 was considered to be sufficient in the tested mediation models comprised of 13 parameters at maximum (i.e., motor coordination, MVPA, and 11 domain-specific PSC factors). Results of missing data analysis showed lower than 5% missing values in all variables (e.g., 0%–1.3% in the ADC, 0%–3.4% in the IPAQ-LF, and 0%–2.1% in the PSDQ-S). Therefore, single imputation with regression substitution was conducted to estimate the missing values. Variables with missing values were entered into a regression model, including body height and weight, time spent in different domains of physical activity, and item scores for all other measures (e.g., ADC and PSDQ-S). Taking into account the potential effect of sex on the variables measured in this study, the missing values were imputed for males and females, separately [29]. 

**Figure 1:**
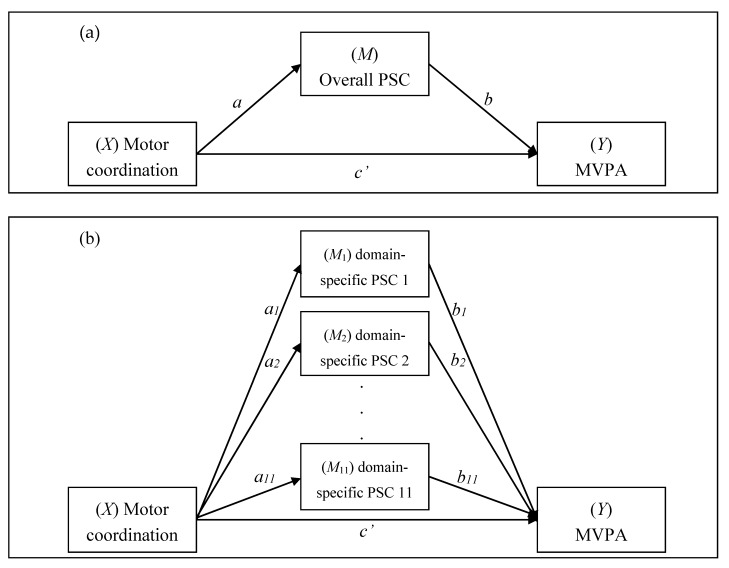
Conceptual diagrams of (**a**) the simple mediation model and (**b**) the parallel multiple mediator model. Note: *a* indicates the unstandardized coefficient from *X* to *M*, *b* indicates the unstandardized coefficient from *M* to *Y*, and *c*’ indicates the unstandardized coefficient from *X* to *Y*. MVPA: moderate-to-vigorous physical activity; PSC: physical self-concept

In order to control for potential bias resulting from outliers, a *z*-score for each variable was computed. All outliers (i.e., *z*-score ≥ 3) were excluded from the analysis. This resulted in a final sample of 218 participants. Descriptive statistics and bivariate (Pearson’s *r*) correlation coefficients were computed for the relationships between motor coordination, PSC, and MVPA. Indirect (mediation) effects were assessed using the PROCESS software macro [30]. Bias-corrected bootstrap procedures with 10,000 simulations were computed, as recommended by Hayes and Scharkow [31]. First, to evaluate the hypothesis that overall PSC mediates the effect of motor coordination on total and subdomain MVPA, indirect effects analyses were computed (Figure 1a). Second, the parallel multiple mediator model was computed to evaluate the mediating effects of each potential domain-specific PSC simultaneously (Figure 1b). However, if a domain-specific PSC was found not to be associated with either motor coordination and MVPA, it was excluded from the examination of the parallel multiple mediator model. Considering the potential statistical bias due to an unequal distribution of sex and a narrow range of age, both variables were controlled for in all tested models in the present study.

Based on recommendations by Hayes [32], an association between *X* (the predictor, i.e., motor coordination) and *Y* (the dependent variable, i.e., MVPA) does not need to exist (as previously suggested by Baron and Kenny) in order to examine the indirect effect of *X* on *Y* through *M* (the mediator, i.e., overall or domain-specific PSC) [32]. In other words, we directly examined the mediating effect of PSC on the relationship between motor coordination and MVPA, and a confidence interval that did not include zero indicated a significant (*p* < 0.05) indirect (mediation) effect [31].

## 3. Results

### 3.1. Descriptive Statistics 

The means and standard deviations or the percentages of all variables are provided in Table 1. The majority of the participants were female (*n* = 167, 76.6%), in the first year of their programs (*n* = 116, 53.5%), and recruited from the School of Science (*n* = 113, 53.1%). There were 22 participants (10.1%) who reported not spending any time engaging in MVPA in the past seven days, while more than 50% (*n* = 126) of participants reported meeting the physical activity guidelines (i.e., MVPA ≥ 150 min/week). 

### 3.2. Correlations between Motor Coordination, PSC, and MVPA

As shown in Table 2, poor motor coordination was moderately associated with lower levels of overall PSC (*r* = −0.463, *p* < 0.001) and most of the domain-specific PSC (*r* = −0.139 to −0.465, all *p*’s were <0.05). However, no significant association between motor coordination and either total MVPA or MVPA subdomains was found (all *p*’s were >0.05). Total MVPA demonstrated a significant positive association with overall PSC (*r* = 0.317, *p* < 0.001), as well as most domain-specific PSC factors (*r* = 0.136 to 0.504, all *p*’s were <0.05). Details of associations between different subdomains of MVPA and PSC are provided in Table 2. 

### 3.3. Mediation of Overall PSC on the Relationship between Motor Coordination and MVPA 

Table 3 shows the results of the single mediation models for the relationship between motor coordination and total MVPA, as well as the subdomains of MVPA through overall PSC. Motor coordination demonstrated a significant indirect effect on total MVPA through overall PSC (effect = −28.72, bootstrap standard error (SE) = 7.04, 95% bootstrap confidence interval (CI) = −43.28, −15.81). Furthermore, when the mediating effects of overall PSC on the relationships between motor coordination and MVPA subdomains were tested, significant indirect effects of motor coordination were also found for chore-related MVPA (effect = −8.29, bootstrap SE = 3.48, 95% bootstrap CI = −15.63, −7.96) and leisure-time MVPA (effect = −16.68, bootstrap SE = 3.60, 95% bootstrap CI = −24.17, −10.14). 

### 3.4. Multiple Mediation of Domain-Specific PSC on the Relationship between Motor Coordination and MVPA 

As the perception of body fat was not associated with total MVPA or any MVPA subdomain, it was excluded from the parallel multiple mediation analyses. Finally, there were five parallel multiple mediator models, with *Y* representing overall MVPA and four MVPA subdomains, respectively (Figure 1b). When domain-specific PSC was tested, while controlling for sex and age (Table 4), the perception of activity was found to significantly mediate the relationship between motor coordination and total MVPA (effect = −25.59, bootstrap SE = 7.67, 95% bootstrap CI = −41.42, −11.32), chore-related MVPA (effect = −4.43, bootstrap SE = 2.70, 95% bootstrap CI = −10.62, −0.18), and leisure-time MVPA (effect = −18.36, bootstrap SE = 5.02, 95% bootstrap CI = −28.53, −8.71). The effects of motor coordination on chore-related MVPA and leisure-time MVPA were also mediated by the perceptions of health (effect = −2.59, bootstrap SE = 1.65, 95% bootstrap CI = −6.37, −0.02) and global esteem (effect = −5.21, bootstrap SE = 2.74, 95% bootstrap CI = −11.40, −0.56), respectively. There was also a significant indirect effect observed of motor coordination on job-related MVPA through the perception of coordination (effect = 11.69, bootstrap SE = 6.45, 95% bootstrap CI = 0.91, 26.19) and flexibility (effect = −6.06, bootstrap SE = 2.91, 95% bootstrap CI = −12.83, −1.44), and on transportation MVPA through the perception of general physical (effect = −2.18, bootstrap SE = 1.53, 95% bootstrap CI = −5.94, −0.12) and endurance (effect = 1.80, bootstrap SE = 1.23, 95% bootstrap CI = 0.04, 4.75). 

## 4. Discussion

This study was the first to comprehensively investigate the complex relationships among motor coordination, overall and domain-specific PSC, and total and subdomain MVPA in emerging adults. Overall, our findings support our hypotheses, indicating that the relationship between motor coordination and MVPA is mediated by PSC. In other words, emerging adults with poor motor coordination exhibit lower PSC, which is subsequently associated with less MVPA. Importantly, these mediating pathways differ by domain-specific PSC and domains of physical activity.

Consistent with prior research showing that children and adolescents with poor motor coordination exhibited lower levels of PSC [16,17], our study extended these findings to emerging adults and demonstrated that lower PSC is also associated with worse motor coordination in this population, suggesting that lower levels of PSC in children and adolescents with poor motor coordination may persist into adulthood. Furthermore, our findings suggest a mediating effect of overall PSC on the relationship between motor coordination and MVPA. In other words, low levels of motor coordination among emerging adults lead to lower levels of PSC which, in turn, lead to lower levels of MVPA. Specifically, this finding suggests that the inclusion of intervention efforts to specifically target PSC in addition to broader physical activity promotion is recommended for emerging adults. 

Although the research examining the impact of motor coordination on MVPA in emerging adults is limited, these findings are consistent with a recent study of adolescents aged 11–17 years. When domain-specific PSC factors, including strength, endurance, coordination, and flexibility, were separately tested by Jekauc and colleagues [18], the mediating effect on the relationship between motor abilities and MVPA was only found for strength, coordination, and flexibility. In our study, when more domains of PSC were simultaneously examined, only activity demonstrated a significant mediating effect, suggesting that the relationships among motor coordination, physical self, and MVPA may be dynamic and change from childhood through adolescence into adulthood. Further longitudinal studies tracking children into adolescence and emerging adulthood are needed to confirm this hypothesis.

A number of studies consistently showed that the perception of perceived motor or athletic competence may mediate the relationship between fundamental motor skills and participation in physical activity in children and adolescents [19,33,34,35]. In other words, motor difficulties result in lower perceived motor competence that further deters from sport participation and physical activity and, in turn, negatively impacts the development of physical fitness, particularly healthy body mass [19,33]. Nevertheless, a different pattern emerges in adulthood; instead of perceptions relating to motor coordination [19,34,35] or physical fitness [18], the perception of physical activity may be a more salient factor that impacts engagement in MVPA in emerging adults with poor motor coordination. Emerging adults with poor motor coordination may view themselves as more physically inactive and could withdraw from MVPA, specifically in relation to chores and leisure-time activities, particularly if they require a higher activity level. However, as this study did not examine differences in the activity levels between MVPA subdomains, further research is needed to confirm this assumption. 

Another novelty of this study was the examination of the mediating effects on specific subdomains of MVPA. While prior research has often considered total physical activity or MVPA as the sole outcome of interest [18,19,34,35], this study investigated different types of MVPA, including job-related, transportation, chores, and leisure-time activities. Our findings showed that the perception of activity seems to be a consistent mediator, and that the pattern for each type of MVPA is different. For example, the perceptions of activity and health were found to mediate the relationship between motor coordination and chore-related MVPA, whereas activity and global esteem were found to mediate the relationship between motor coordination and leisure-time MVPA. Furthermore, despite overall PSC not mediating the relationships between motor coordination and either job-related or transportation MVPA, the relationships are mediated by other unique domain-specific PSC factors in this study. As different subdomains of MVPA may bring different benefits, in particular, leisure-time physical activity and sport that may be more associated with physical or mental health [22], specific intervening strategies should be developed based on the targeted areas of physical activity. It is also worth noting that, as far as we are aware, this study was the first to explore the complexity of these relationships, yet the underlying mechanisms are still unclear and more research is warranted to support our findings, specifically in emerging adults. 

Our findings have significant practical implications for enhancing physical activity levels in emerging adults, specifically undergraduate students, for which physical activity levels are alarmingly low at 34% according to recent reports [36]. In addition to improving motor coordination or skills essential for MVPA, our findings for the mediation of PSC suggest that interventions should target improvements in PSC in university students with poor motor coordination. This could be accomplished by utilizing some specific strategies, for example, recreation-based physical education classes or sport clubs should be appropriately classified into different levels (e.g., fundamental, intermediate, or skilled) based on students’ motor coordination, skills, or previous experience. By matching opportunities for participation to ability level, positive PSC could result through supportive, positive environments. This may be particularly beneficial for those students who may have been previously physically inactive. Otherwise, participation in sport activities could conversely cause an adverse effect on their PSC, such as a sense of failure [37,38], which may result in a negative experience and consequently hinder them from regular physical activity going forward. 

While findings from this study shed light on the potential impact of PSC on MVPA in university students with poor motor coordination, there are a few limitations that need to be considered. For example, there was an unequal sample size between male and female participants, which may have limited our ability to examine the potential sex effect on the relationships of interest in this study. Second, as more than 50% of participants met the recommended amount of daily MVPA in this study, which is higher than the national prevalence, we may have recruited more physically active emerging adults. Furthermore, a majority of our participants were first-year undergraduate students who were more likely to live on campus, potentially limiting the generalizability of our results to the broader emerging adulthood population. Third, as a self-reported questionnaire of physical activity was used in this study, physical activity may have been overestimated by our participants. It is important to re-visit this issue using objective measures (e.g., accelerometers) in future work. Furthermore, as our participants were all undergraduate students, they likely spent limited time on job-related physical activity, which may have biased our findings. Finally, this study was cross-sectional in design. Future longitudinal studies would allow us to examine changes in the identified relationships over time and provide more robust evidence regarding causality between motor coordination, PSC, and MVPA. 

## 5. Conclusions

This study highlighted that the relationship between motor coordination and moderate-to-vigorous physical activity is mediated by physical self-concept. That is, emerging adults with poor motor coordination exhibit lower levels of PSC that subsequently impact their willingness to participate in moderate-to-vigorous physical activities. Furthermore, the perception of physical activity may be a more important factor when targeting improvement in participation in moderate-to-vigorous physical activity in university students with poor motor coordination. 

## Figures and Tables

**Table 1:** Descriptive statistics of the sample (*n* = 218).

Variables	Mean ± SD or *n* (%)	Range
Age (years)	19.5 ± 1.0	17.3–23.3
Female	167 (76.6%)	
University year ^a^		
First	116 (53.5%)	
Second	63 (29.0%)	
Third	28 (12.9%)	
Fourth	10 (4.6%)	
Faculty ^b^		
Business	15 (7.0%)	
Engineering	22 (10.3%)	
Health Science	25 (11.7%)	
Humanities	9 (4.2%)	
Science	113 (53.1%)	
Social Science	26 (12.2%)	
Other	3 (1.4%)	
Motor coordination	47.6 ± 10.2	30–84
MVPA (MET-min/week)		
Job-related	278.7 ± 1045.4	0–6480.0
Transportation	70.0 ± 461.8	0–5040.0
Chores	620.5 ± 1016.6	0–6630.0
Leisure-time	725.6 ± 1136.6	0–6720.0
Total	1694.8 ± 1936.8	0–8910.0
Overall PSC	3.9 ± 0.8	1.9–5.8
Domain-specific PSC		
Health	4.7 ± 1.0	1.4–6.0
Coordination	4.2 ± 1.1	1.0–6.0
Activity	3.3 ± 1.4	1.0–6.0
Body fat	4.1 ± 1.5	1.0–6.0
Sport	3.5 ± 1.5	1.0–6.0
Global physical	3.6 ± 1.4	1.0–6.0
Appearance	3.5 ± 1.2	1.0–6.0
Strength	3.7 ± 1.2	1.0–6.0
Flexibility	3.6 ± 1.4	1.0–6.0
Endurance	3.3 ± 1.3	1.0–6.0
Global esteem	4.4 ± 1.0	1.6–6.0

^a^ Missing data in one participant; ^b^ missing data in five participants. MVPA: moderate-to-vigorous physical activity; PSC: physical self-concept.

**Table 2:** Correlations between motor coordination, PSC, and MVPA.

PSC	Motor Coordination	MVPA				
		Job-Related	Transportation	Chores	Leisure-Time	Total
**Domain-specific**						
Health	−0.245 ***	0.035	0.024	0.112	0.020	0.095
Coordination	−0.465 ***	0.013	0.010	0.130	0.113	0.144 *
Activity	−0.275 ***	0.126	0.030	0.196 **	0.555 ***	0.504 ***
Body fat	−0.090	−0.058	−0.052	0.097	−0.035	−0.013
Sport	−0.397 ***	0.099	−0.027	0.138 *	0.167 *	0.218 **
Global physical	−0.308 ***	0.022	0.026	0.120	0.093	0.136 *
Appearance	−0.139 *	0.032	−0.041	0.049	0.042	0.058
Strength	−0.411 ***	0.137 *	0.013	0.118	0.290 ***	0.309 ***
Flexibility	−0.297 ***	0.178 **	−0.011	0.092	0.138 *	0.223 **
Endurance	−0.284 ***	0.096	−0.051	0.167 *	0.312 ***	0.310 ***
Global esteem	−0.313 ***	0.058	−0.037	0.108	0.187 **	0.189 **
**Overall**	−0.463 ***	0.103	−0.013	0.193 **	0.279 ***	0.317 ***

* *p* < 0.05; ** *p* < 0.01; *** *p* < 0.001. MVPA: moderate-to-vigorous physical activity; PSC: physical self-concept.

**Table 3:** The mediating effects of overall PSC.

*Y* (*M* = overall PSC)	Effect	Bootstrap SE	95% Bootstrap Confidence Interval	
			Lower Limit	Upper Limit
Job-related MVPA	−3.65	3.33	−10.30	3.00
Transportation PA	−0.10	2.51	−4.87	5.12
Chore-related VPA	−***8.29***	***3.48***	−***15.63***	−***7.96***
Leisure-time VPA	−***16.68***	***3.60***	−***24.17***	−***10.14***
Total MVPA	−***28.72***	***7.04***	−***43.28***	−***15.81***

Note. ***Bold*** indicates a significant mediating effect. MVPA: moderate-to-vigorous physical activity; PSC: physical self-concept, SE: standard error

**Table 4:** The multiple mediating effects of domain-specific PSC.

*M*	*Y*				
	Total MVPA	Job-Related MVPA	Transportation MVPA	Chore-Related MVPA	Leisure-Time MVPA
Health	−2.54 (3.03) [−9.26, 2.94]	0.02 (1.72) [−3.35, 3.66]	−0.68 (1.30) [−3.73, 1.41]	−***2.59 (1.65) [***−***6.37,*** −***0.02]***	0.71 (1.39) [−2.06, 3.69]
Coordination	14.36 (8.52) [−2.17, 31.67]	***11.69 (6.45) [0.91, 26.19]***	−0.96 (1.67) [−4.74, 1.92]	−2.12 (3.88) [−10.48, 5.03]	5.75 (5.01) [−3.28, 16.53]
Activity	−***25.59 (7.67) [***−***41.42,*** −***11.32]***	−1.33 (2.20) [−6.02, 2.88]	−1.48 (1.18) [−4.39, 0.12]	−***4.43 (2.70) [***−***10.62,*** −***0.18]***	−***18.36 (5.02) [***−***28.53,*** −***8.71]***
General physical	2.73 (5.28) [−7.78, 13.41]	3.18 (2.74) [−1.29, 9.51]	−***2.18 (1.53) [***−***5.94,*** −***0.12]***	−2.41 (3.65) [−10.25, 4.17]	4.14 (3.12) [−1.76, 10.64]
Flexibility	−7.30 (4.45) [−17.18, 0.27]	−***6.06 (2.91) [***−***12.83,*** −***1.44]***	0.23 (1.07) [−1.93, 2.41]	−0.68 (2.19) [−5.33, 3.39]	−0.79 (2.10) [−4.82, 3.58]
Endurance	1.36 (3.77) [−6.15, 9.06]	0.74 (2.05) [−3.05, 5.28]	***1.80 (1.23) [0.04, 4.75]***	−1.68 (2.26) [−6.51, 2.54]	0.51 (2.41) [−4.59, 5.07]
Global esteem	−4.18 (5.19) [−15.16, 5.66]	−0.61 (2.47) [−5.81, 4.17]	1.36 (2.15) [−1.70, 6.34]	0.28 (2.98) [−5.58, 6.40]	−***5.21 (2.74) [***−***11.40,*** −***0.56]***

Note. Effects (bootstrap SE) (95% bootstrap confidence interval) are reported for each mediator. ***Bold*** indicates a significant mediating effect. As there were no significant mediating effects observed regarding the perceptions of sport, appearance, and strength on the relationship between motor coordination and MVPA subdomains, they do not appear in this table. MVPA: moderate-to-vigorous physical activity; PSC: physical self-concept.
